# First report of *Meloidogyne enterolobii* infecting Japanese blue berry tree (*Elaeocarpus decipiens*) in Florida, USA

**DOI:** 10.21307/jofnem-2020-005

**Published:** 2020-03-06

**Authors:** M. R. Moore, J. A. Brito, S. Qiu, C. G. Roberts, L. A. Combee

**Affiliations:** 1 Molecular Diagnostics Laboratory, Florida Department of Agriculture and Consumer Services, Division of Plant Industry, Gainesville, FL 32608.; 2 Nematode Diagnostic Laboratory, Florida Department of Agriculture and Consumer Services, Division of Plant Industry, Gainesville, FL 32608.

**Keywords:** Elaeocarpaceae, *Elaeocarpus decipiens*, Guava root-knot nematode, Japanese blueberry tree, *Meloidogyne enterolobii*, Pacara earpod tree root-knot nematode, Regulatory

## Abstract

In October 2019, samples of galled roots with rhizosphere soil were collected from declining *Elaeocarpus decipiens* in Hernando County, Florida. Extracted root-knot nematodes were identified by both molecular and morphological methods as *Meloidogyne enterolobii*. This is a first report of this regulated root-knot nematode on *Elaeocarpus decipiens* in Florida.

*Elaeocarpus decipiens* F.B.Forbes & Hemsl. (Japanese blueberry tree; Oxalidales: Elaeocarpaceae) is an evergreen tree native to East Asia. Its stylish branching pattern, opulent growth, solid form and beautiful tropical foliage make this plant species an attractive landscape tree. In October 2019, a sample of soil and roots was collected from under an *E. decipiens* in Hernando County, FL and submitted for certification for *Meloidogyne enterolobii* (Yang and Eisenback, 1983) at the Division of Plant Industry, Florida Department of Agriculture and Consumer Services, Gainesville, FL (FDACS-DPI). This nematode species currently is under quarantine regulations in three states (Arkansas, Louisiana and Mississippi) in the USA.

Nematodes were extracted from both soil and roots and species identification was performed using FDACS-DPI’s standard protocol for *M*. *enterolobii*, a COI-based qPCR assay (Kiewnick et al., 2015; Braun-Kiewnick et al., 2016) with slight modifications: Applied Biosystems QuantStudio 5 platform and SensiFast Lo-Rox chemistry, 40 PCR cycles instead of 45 cycles, annealing time of 30 s instead of 60 s, and an IDT produced custom oligonucleotide positive control is used instead of pure extracted *M*. *enterolobii*. Results of the analysis demonstrated positive identification of *M. enterolobii* in the sample. To determine whether *E. decipiens* is indeed a host of *M*. *enterolobii*, rather than weeds growing together in the pot with this evergreen, additional soil and root samples (*n*=3) were collected directly from the rhizosphere of *E. decipiens*. These samples were designated with the internal FDACS-DPI sample identifier N19-1242. Galls were observed on secondary and tertiary roots (Fig. 1). Females were found inside of the galls, and egg masses were outside.

**Fig. 1:**
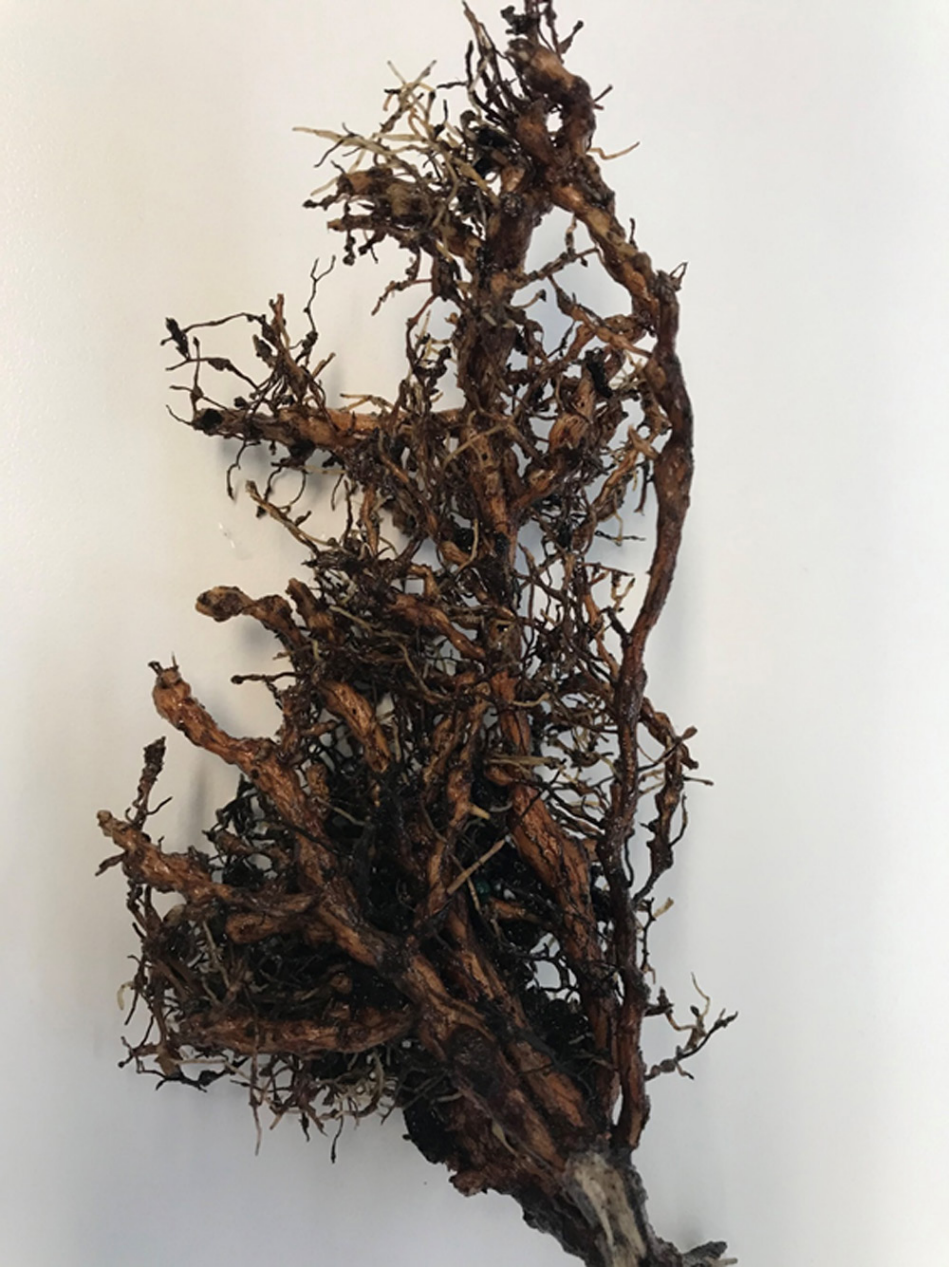
Roots of *Elaeocarpus decipiens* F.B.Forbes & Hemsl. showing galls induced by Meloidogyne enterolobii (Yang and Eisenback, 1983).

Nematode species were identified using molecular and isozyme analyses, body length of second-stage juveniles (J2), and morphology of the perineal patterns. Nematodes (J2) were extracted from soil for DNA extraction according to Holterman et al. (2006). DNA samples were screened for *M. enterolobii* as above. The COI-based qPCR assay was then repeated for positive samples, but with J2 extracted directly from roots. To confirm the results for *M. enterolobii* positive samples, we also performed the IGS2-based qPCR assay (Kiewnick et al., 2015; Braun-Kiewnick et al., 2016) with the same conditions described above, except without a synthetic oligonucleotide positive control. Additionally, we obtained DNA from J2 individuals reared from egg masses using the Qiagen DNeasy Blood and Tissue Kit (Qiagen®, Hilden, Germany) for conventional PCR and sequencing.

Standard PCRs targeted COII using the primers COX2F/COX2R and thermocycle conditions described by Janssen et al. (2016). Purified PCR products were sequenced bidirectionally on an Applied Biosystems SeqStudio platform with BigDye Terminator v. 3.1 cycle sequencing chemistry (Applied Biosystems, Foster City, California). Chromatograms were trimmed and assembled into sequence contigs in Sequencer 5.4.6 (Gene Codes Corporation, Ann Arbor, Michigan). Newly generated sequences (MN842265–MN842267) were aligned in MEGA7 (Kumar et al., 2016) using the default settings of MUSCLE (Edgar, 2004). The new sequences were compared to the corresponding GenBank COII “popset” (PopSet: 1005136704) generated by Janssen et al. (2016) and K2P (Kimura 1980) neighbor-joining analysis with complete deletion of missing data. Additionally, SCAR PCRs using species-specific primers MK7-F/MK7-R (Tigano et al., 2010) were used to further confirm the identity of N19-1242.

The COI-based qPCR assay yielded Ct values of 24.061–34.011 (*n*=13). The IGS2-based qPCR assay yielded Ct values of 23.789–24.975 (*n*=3). COII sequences were 100% matches to previously reported *M*. *enterolobii* data based on BLASTn searches and neighbor-joining analysis (Fig. 2). The SCAR PCR was positive for *M*. *enterolobii* (Fig. 3), yielding the predicted 520 bp product (Tigano et al., 2010). Isozyme analysis (EST=VS1-S1; MDH=N1a) of females (*n*=26) were identical to earlier reports of this nematode species (Brito et al., 2004). Perineal patterns of females (*n*=20) and J2 body length (*n*=18) were consistent with the original description of *M. enterolobii* (Yang and Eisenback, 1983). To our knowledge this is the first report of *E. decipiens* as a host of *M. enterolobii* in Florida.

**Fig. 2:**
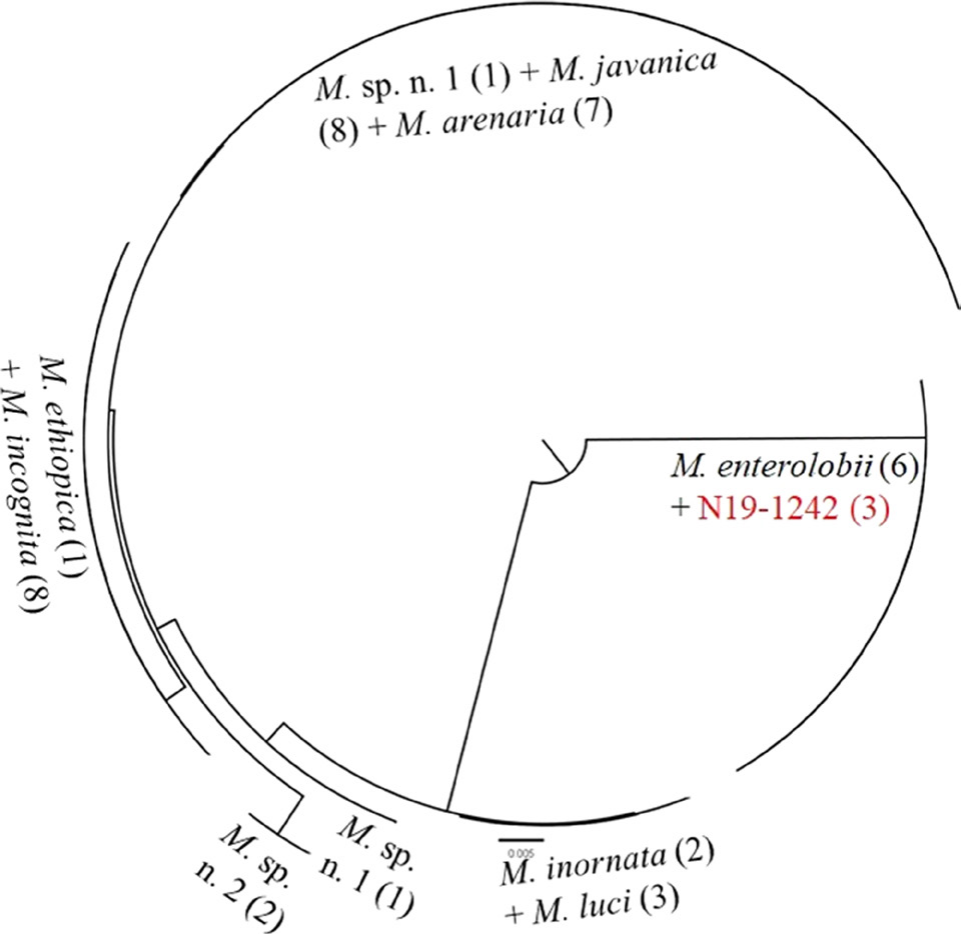
K2P neighbor-joining tree depicting the clustering of COII sequences. Samples collected from *Elaeocarpus decipiens* F.B.Forbes & Hemsl. in Florida are highlighted in red. Numbers in parentheses indicate sequences per nematode species.

**Fig. 3:**
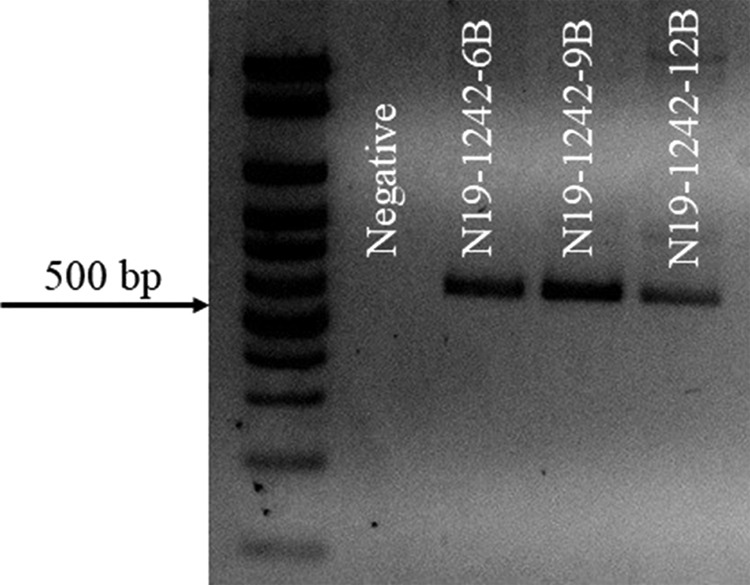
PCR amplification products of DNA extracted from *Meloidogyne enterolobii* (Yang and Eisenback, 1983) using mtDNA primer set MK7-F/MK7-R SCAR. Negative control=PCR reagents without DNA. Low range DNA ladder 2,000 bp.
